# A Good Prescription

**Published:** 1887-07-23

**Authors:** 


### A GOOD PRESCRIPTION.

Some time ago a lad swallowed a small leaden bullet. His friends were very much alarmed about it. A doctor was found, heard the dismal tale, and, with as much unconcern as he would have manifested in a case of common headache, wrote the following laconic note to the lad's father: "Sir,—Don't alarm yourself. If after three weeks the bullet is not removed, give the boy a charge of powder.—Yours, etc., etc. P.S.—Don't shoot the boy at anybody."

